# Predicting the risk of a clinical event using longitudinal data: the generalized landmark analysis

**DOI:** 10.1186/s12874-022-01828-x

**Published:** 2023-01-07

**Authors:** Yi Yao, Liang Li, Brad Astor, Wei Yang, Tom Greene

**Affiliations:** 1 Department of Biostatistics, University of Texas MD Anderson Cancer Center, Houston, TX, US; 2 School of Medicine and Public Health, University of Wisconsin-Madison, Madison, WI, US; 3 Perelman School of Medicine, University of Pennsylvania, Philadelphia, PA, US; 4 School of Medicine, University of Utah, Madison, UT, US

**Keywords:** Chronic kidney disease, Dynamic prediction, Landmarking analysis, Longitudinal data analysis, Survival analysis

## Abstract

**Background:**

In the development of prediction models for a clinical event, it is common to use the static prediction modeling (SPM), a regression model that relates baseline predictors to the time to event. In many situations, the data used in training and validation are from longitudinal studies, where predictor variables are time-varying and measured at clinical visits. But these data are not used in SPM. The landmark analysis (LA), previously proposed for dynamic prediction with longitudinal data, has interpretational difficulty when the baseline is not a risk-changing clinical milestone, as is often the case in observational studies of chronic disease without intervention.

**Methods:**

This paper studies the generalized landmark analysis (GLA), a statistical framework to develop prediction models for longitudinal data. The GLA includes the LA as a special case, and generalizes it to situations where the baseline is not a risk-changing clinical milestone with a more useful interpretation. Unlike the LA, the landmark variable does not have to be time since baseline in the GLA, but can be any time-varying prognostic variable. The GLA can also be viewed as a longitudinal generalization of localized prediction, which has been studied in the context of low-dimensional cross-sectional data. We studied the GLA using data from the Chronic Renal Insufficiency Cohort (CRIC) Study and the Wisconsin Allograft Replacement Database (WisARD) and compared the prediction performance of SPM and GLA.

**Results:**

In various validation populations from longitudinal data, the GLA generally had similarly or better predictive performance than SPM, with notable improvement being seen when the validation population deviated from the baseline population. The GLA also demonstrated similar or better predictive performance than LA, due to its more general model specification.

**Conclusions:**

GLA is a generalization of the LA such that the landmark variable does not have to be the time since baseline. It has better interpretation when the baseline is not a risk-changing clinical milestone. The GLA is more adaptive to the validation population than SPM and is more flexible than LA, which may help produce more accurate prediction.

## Introduction

### Risk prediction in longitudinal data

It is of interest to scientific research and clinical practice to develop prediction models for the probability of a terminal clinical event at a future time point (prediction horizon) using prognostic variables of the patients’ health conditions. In many situations, such work is carried out with data from longitudinal cohort studies. In these datasets, patients have repeated clinical visits from study entry to have their health conditions assessed, including patient reported measures, clinical evaluation, diagnostic tests, etc. The end of the follow-up is marked by the terminal clinical event or the time of censoring.

### Static prediction model

Conventionally, risk prediction models are developed from regression of the clinical event outcome on baseline predictor variables, though the predictor variables quantifying the individual’s health conditions are often time-varying and may also be measured after baseline in the longitudinal cohort study data. Such a time-to-event analysis, which uses only a snapshot of the predictor variables at a fixed time point to quantify the risk of the event at prediction horizon is termed static prediction modeling (SPM) in this paper.

The SPM has limitations in the context of longitudinal data. First, the prediction model developed from baseline population may not apply to the population at-risk of the clinical event after a period of follow-up, as a result of the systematic difference between those who remain at-risk and those who experience the clinical event early and hence drop out from the at-risk population. Furthermore, in chronic disease studies, the occurrence of the clinical event may be many years later than the baseline, which could attenuate the association between baseline predictors and the outcome if the predictors vary over time with the patient’s health condition. The longitudinal data from the follow-up visits are temporally closer to the clinical event and hence informative, but they are ignored in SPM.

### Dynamic prediction with landmark analysis

Dynamic prediction modeling is specifically developed for risk prediction in the longitudinal context [1, 2]. There are generally two approaches: joint modeling [3, 4] and landmark analysis [5, 6]. The features and relative advantages of these two modeling approaches have been widely discussed in the literature (e.g., [7–10]). In this paper, we focus on landmark analysis (LA), and propose a generalization of it. LA directly models the bilateral relationship between predictor variables at each clinical visit and the corresponding residual survival time through a survival regression model fitted to training data. The time of each clinical visit is called a landmark time because it defines a predictor-outcome pair and marks the start of the residual survival for patients who remain at risk for the outcome at the index visit. Once this model is estimated, for any new patient in the validation dataset who needs a prognosis during a clinical visit, the physician can elicit the predictor variables corresponding to that visit, plug into the regression model, and obtain the prediction. The estimation of the LA model can often be implemented with standard survival regression analysis software.

### Generalizing the landmark analysis

A critical concept in the LA is the baseline, because its typical parameterization formulates the model parameter as a function of the time since baseline [1, 6, 7, 11, 12]. We denote the model by $\mathcal {M}(\varvec{\theta }(s))$, where *s* is the time after baseline and the vector $\varvec{\theta }(s)$ includes all model parameters. Any appropriate survival regression models can be used. With a slight abuse of notation, we also use $\mathcal {M}$ to denote the predicted residual survival distribution, and write it as $\mathcal {M}(\varvec{\theta }(s); \varvec{Z})$, where we add the predictor $\varvec{Z}$ at the clinical visit where a prediction is to be made, to show that the prediction is a function of the predictor. As an example, consider a prediction made at time *s* from a Cox model. Let $\varvec{\theta }(s)$ cover parameters in the baseline hazard function $\lambda _0(.)$, denoted by $\varvec{\theta }_1(s)$, and regression coefficients, denoted by $\varvec{\theta }_2(s)$. Then, the cumulative distribution function of the predicted residual survival is $1 - \textrm{exp}\left\{ -\int _0^t \lambda _0(u; \varvec{\theta }_1(s))du\cdot \textrm{exp}[ \varvec{Z}^T\varvec{\theta }_2(s)] \right\}$, $t \geqslant 0$. The proposed notation $\mathcal {M}(\varvec{\theta }(s); \varvec{Z})$ denotes this distribution function, which is the basis of survival prediction.

The index *s* is the landmark time. It is an interpretable and clinically meaningful quantity when the baseline time represents a risk change event or clinical milestone, such as initiation of a new intervention or diagnosis of a disease. However, this is not always the case in clinical research.

In this paper, we present two data applications. The first data application is a longitudinal observational cohort study on chronic kidney disease (CKD). The baseline is the enrollment of patients with existing CKD. Each enrolled patient underwent annual follow-up visits until death or end stage renal disease (ESRD). The goal is to predict the individual risk of ESRD at each study visit, using the available longitudinal information about a patient by that time. Since CKD is a chronic disease and the observational study does not provide any intervention, it is difficult to justify indexing the prediction models with the time since enrollment. If the prediction model is applied to patients in another study or healthcare facility, the “time since enrollment” is not defined or uniquely justified. Therefore, for the CKD data application, the conventional LA model with the parameterization above is difficult to apply or communicate to clinicians. For comparison purposes, we also present a second data application from a kidney transplantation registry. The baseline (study entry) is the time of kidney transplant surgery. The transplant recipients are followed until death or graft failure. The goal is to use the available longitudinal information to predict the risk of graft failure among those who are still at-risk of graft failure at any time *s* after the surgery. If we want to make a prediction on any new patient, we first find out how many months (e.g., *s*) the patient has lived with the functioning graft, and then gather the predictor $\varvec{Z}$ for that patient and plug it in the LA model. Here *s* is a clinically meaningful quantity and, since the at-risk patient population is expected to change over time, it makes sense to allow the model parameters to vary with *s*. This is the typical data setting where conventional LA is applicable.

This paper proposes the generalized landmark analysis (GLA) as an alternative parameterization of the LA model, where the model parameters do not need to be functions of the time since baseline, but can be functions of other time-varying variables. Hence, the concept of “landmark time” is generalized to “landmark variable”, whose definition does not rely on a clinically meaningful baseline and applies to the CKD data application. Similar to the above, we denote this parameterization by $\mathcal {M}( \varvec{\theta }(\varvec{V}); \varvec{Z} )$ where $\varvec{V}$ is the landmark variable that indexes the model parameters and $\varvec{Z}$ is the predictor variable at the clinical visit when a prediction is to be made. We argue that it is more beneficial to pick a strong predictor of the survival outcome as the landmark variable, analogously to choosing time since baseline as the landmark variable in an LA analysis when the baseline is a clinical milestone. The GLA is analogous to LA in many ways, and includes the LA as a special case. Details will be explained in the subsequent sections. This parameterization leads to a local kernel weighting procedure for estimation, where larger weights are assigned to subjects in the training dataset who are more similar to the new subject for whom a prediction is to be made. When the kernel function is uniform, it is equivalent to matching on $\varvec{V}$ with a caliper that equals the kernel bandwidth. From this perspective, the GLA can also be viewed as a matching algorithm or a generalization of the “localized” regression of Kosel and Heagerty [13], which was developed for cross-sectional data, to longitudinal context.

## Method

### Data and Notation

Let $i=1,2,...,n$ index the *n* subjects in a longitudinal dataset for training the prediction model. Let $\tilde{T}_{i}$ be the time to the clinical endpoint of interest, and $C_{i}$ be the time to censoring. The observed time-to-event is $T_{i} = {\textrm{min}}\{ \tilde{T}_{i}, C_{i} \}$. The observed event indicator is $\delta _{i} = 1\{ \tilde{T}_{i} \leqslant C_{i} \}$, which equals to 1 if the event occurrence is observed and 0 if the event is censored. We make the conventional independent censoring assumption that $C_{i}$ is independent of $\tilde{T}_{i}$ and predictor variables. This assumption is valid in studies where the censoring is mainly due to staggered study entry or loss of follow-up due to non-medical reasons. Let $t_{ij}$, $j = 1, ..., n_{i}$, index the clinical visit times of the *i*-th subject. The patient’s characteristics and health conditions are measured at these visits. Throughout this paper, we assume that $\{ t_{ij} \}$ are non-informative observation times in the sense that their distribution does not depend on other variables except $0 \leqslant t_{ij} \leqslant T_i$. Without loss of generality, we let $0 \equiv t_{i1}< t_{i2}< ...< t_{in_i} < T_i$. Here $t_{i1}$ is the time of baseline, which may be study entry or enrollment, receipt of study intervention, diagnosis of a disease condition, etc., depending on the context. As described above, this formulation is most useful if the baseline represents a clinical milestone, but this is not required. Unless stated otherwise, all time variables above are expressed as the time since $t_{i1}$.

Let $\varvec{Z}_i(t_{ij})$ denote subject *i*’s vector of predictors at time $t_{ij}$. For the ease of notation, let $\varvec{Z}_{ij} \equiv \varvec{Z}_i(t_{ij})$. This notation covers both time-varying and time-invariant variables. For the latter, $\varvec{Z}_{ij} \equiv \varvec{Z}_{ij'}$ for any $j \ne j'$. The predictor vector $\varvec{Z}_{ij}$ may include any predictive numerical features of patient *i* available at time $t_{ij}$, including biomarkers from lab tests, clinical symptoms, patient demographics and genetic features, etc. $\varvec{Z}_{ij}$ may also include any pre-defined numerical features of the longitudinal history of this patient up to $t_{ij}$, such as the mean, slope or volatility of a biomarker within a period of time prior to $t_{ij}$. One or more of the three time variables ($t_{ij}$, the baseline age, and the time-varying age at $t_{ij}$) should always be included in $\varvec{Z}_{ij}$ in the absence of exact collinearity; otherwise the timing of the longitudinal data is lost. Note that the age at $t_{ij}$ equals to the baseline age plus $t_{ij}$ in a deterministic relationship, and baseline age is just the time-varying age at $t_{i1}$.

Let $\tilde{T}_{ij} = \tilde{T}_i - t_{ij}$ and $C_{ij} = C_i - t_{ij}$ be the residual survival and residual censoring time since the clincial visit at $t_{ij}$. $\tilde{T}_{ij}$ and $C_{ij}$ are conditionally independent given $\varvec{Z}_{ij}$, due to the independent censoring assumption. The observed residual survival is hence $T_{ij} \equiv T_i(t_{ij}) = min\left( \tilde{T}_{ij}, C_{ij} \right) = T_{i} - t_{ij}$. The observed residual censoring indicator is $\delta _{ij} \equiv \delta _i(t_{ij}) = \textbf{1}\left( \tilde{T}_{ij} \le C_{ij} \right) = \delta _i$. The landmark dataset for training purpose has $\sum _{i=1}^n n_i$ rows. At each row, the data include $T_{ij}$, $\delta _{ij}$, and $\{ t_{ij} \} \cup \{ \varvec{Z}_{ij} \}$. By definition, patient *i* is at-risk at the *j*-th visit, if the corresponding $t_{ij}$ is in the landmark dataset. The goal of the landmark analysis is to build a model for the bilateral relationship between $\varvec{Z}_{ij}$ and $\tilde{T}_{ij}$ from the landmark dataset. For a new patient in validation data, we first determine the $\varvec{Z}$ of that patient at the time of prediction, and then plug the $\varvec{Z}$ in the model to obtain the prediction.

### Model parameterization for landmark analysis

The LA model takes the following general form, specified based on the Cox model formulation:

1 $$\begin{aligned} \lambda (u; \varvec{Z}(s)) = \lambda _0(u, s) + \varvec{\beta }(u, s)^T \bar{\varvec{Z}}(s)~~, ~~~~ u \geqslant 0. \end{aligned}$$

This is a log hazard function of the residual survival since the landmark time *s*, conditional on $\varvec{Z}(s)$. The notation *u* denotes time since the landmark, and hence is on the residual survival time scale. The $\lambda _0(u, s)$ is the log baseline hazard function, and $\varvec{\beta }(u, s)$ is the time-varying log hazard ratios. We use notation $\bar{\varvec{Z}}(s)$ to denote the vector of predictor variables measured at *s* other than the landmark time *s* itself. The effect of *s* is absorbed into $\lambda _0(u, s)$. This model is specified among patients who have $\varvec{Z}(s)$, i.e., who are at-risk of the event outcome. At a given *s*, (1) is a Cox model with time-independent covariates and time-varying coefficients. But since *s* can be any landmark time, the LA models as a whole include a bivariate log baseline hazard function, which is quite different from Cox model. The LA models at different landmark times do not generally satisfy the coherent condition of a survival process [14]. Consequently, $\lambda (s_2 - s_1; \varvec{Z}(s_1)) \ne \lambda (0; \varvec{Z}(s_2))$ for all $s_1 < s_2$. Therefore, (1) is usually viewed as a working model [1]. However, as far as prediction is concerned, this model is useful if it ensures a good approximation to the bilateral relationship between $\varvec{Z}_{ij}$ and $\tilde{T}_{ij}$ at all landmark times. In other words, the model predicted survival distribution $1 - \textrm{exp}\{ -\int _0^u \lambda (v; \varvec{Z}(s)) dv \}$ needs to match the observed residual survival data. While most LA research has used a Cox model-based formulation such as (1), other survival regression models can be used [15]. The previously introduced notation $\varvec{\theta }(s)$ encompasses all the model parameters at landmark time *s*, including $\varvec{\beta }(u, s)$ and $\lambda _0(u, s)$.

Model (1) has never been applied in its fully general form in the published literature, possibly because of the excessive number of parameters if all coefficients are bivariate functions of time. Simplified versions used in data analysis include: (A) letting $\varvec{\beta }(u, s)$ to depend only on *s* [1, 7, 12], (B) letting $\varvec{\beta }(u, s)$ depend only on *u* [6, 16], and (C) letting $\lambda _0(u, s)$ to be a product of two univariate functions of *u* and *s* [17]. Regardless of these parameterizations, the LA model (1) implies infinitely many survival models, each defined on a distinct *s* and with parameter $\varvec{\theta }(s)$. It should not be viewed as a single varying-coefficient model with bivariate time-varying coefficients, because there are infinitely many values of *s* as well as a second argument *u* which corresponds to the time since each *s*. Note that *u* is on the residual survival time scale and hence the model parameters at the landmark time *s* do not depend on *u*. The parameterization (B) was proposed to deal with violation of the proportional hazard assumption. Since the prediction horizon is usually not very large, because of repeated predictions at clinical visits, the log hazard ratio often does not vary substantially within the horizon. Therefore, in this paper, we focus the discussion on the parameterization (A):

2 $$\begin{aligned} \lambda (u; \varvec{Z}(s)) = \lambda _0(u, s) + \varvec{\beta }(s)^T \bar{\varvec{Z}}(s) . \end{aligned}$$

This parameterization can be estimated by kernel weighting [7], where both $\lambda _0(u, s)$ and $\varvec{\beta }(s)$ are estimated nonparametrically. In theory (1) can also be estimated this way. The intuitive idea is to assign kernel weights to the rows of the landmark dataset whose $t_{ij}$ is close to *s*, and estimate $\varvec{\theta }(s)$ by fitting a Cox model with time-independent covariates to the weighted landmark data. Since a single subject may contribute multiple rows with positive weights, this is a Cox model for multivariate survival data with working independence, which is commonly used in landmark analysis research [1, 7, 15]. In this paper, we say this approach “localizes” on the landmark time $\{ t_{ij} \}$. When the clinical visit times of different subjects are synchronized (i.e., $t_{ij} = t_{j}$, such as discrete time points), the kernel approach reduces to just fitting a Cox model at each distinct landmark time [12]. When a uniform kernel function is used, weighting is equivalent to matching: a weight of 1 indicates being matched and a weight of 0 indicates being unmatched. For any new subject in the validation dataset, we search for the training landmark dataset and identify rows with similar landmark times; a Cox model is fit to the matched landmark dataset and a prediction is generated based on the new subject’s predictor $\varvec{Z}$.

In summary, the commonly used LA model (1) is a parameterization for the bilateral relationship between $\varvec{Z}_{ij}$ and $\tilde{T}_{ij}$ in the landmark dataset. This parameterization implies a distinct survival regression model at each landmark time *s*. This model can be estimated by a kernel weighting method that localizes on the landmark time. An algorithmic approach can also be used, where we match the new subject with the landmark dataset on the landmark time, fit a regression model to the matched data, and plug in $\varvec{Z}$ to that model to obtain the prediction.

### A different model parameterization: the generalized landmark analysis

When the baseline is not a clinically meaningful milestone, the time since baseline, i.e., the landmark time *s*, may not be a useful index of the model or parameters with good interpretation. In this paper, we propose a different parameterization for this scenario. The matching interpretation to the estimation approach to model (1) suggests that instead of matching on the landmark time, we could match on another time-varying predictor variable which better reflects disease severity or stage, denoted by $\varvec{V}$. We call $\varvec{V}$ a landmark variable. It is part of $\varvec{Z}$. A survival regression model can then be fit to the matched landmark dataset and produce the prediction.

In lieu of the connection between matching and weighting, we can also use a kernel weighting approach. Let $\varvec{V}^*$ be the landmark variable of a new subject in the validation dataset. The weights of the rows in the landmark training dataset are calculated by a kernel function $K_h$ with bandwidth *h*: $W_{ij} = K_h(\varvec{V}^*, \varvec{V}_{ij})$. For example, $K_h(u) = (4h)^{-1}(1-u^2)\textbf{1}(|u|\le 1)$ is the Epanechnikov kernel. Other kernel function or local linear approximation [18] can also be used. Local polynomial theory suggests that the choice of kernel function and degree of the polynomial are less important than the bandwidth. For simplicity, we use kernel, i.e., local constant, approach. Bandwidth is a tuning parameter of the algorithm and will be discussed later.

Taking one step further, we can view this kernel weighting method as the estimation approach to the following parameterization of the bilateral relationship between $\varvec{Z}_{ij}$ and $\tilde{T}_{ij}$ in the landmark training dataset:

3 $$\begin{aligned} \lambda (u; \varvec{Z}(s)) = \lambda _0(u, \varvec{V}(s)) + \varvec{\beta }(\varvec{V}(s))^T \bar{\varvec{Z}}(s)~~, ~~~~ u \geqslant 0 \end{aligned}$$

Similar to (1), $\bar{\varvec{Z}}(s)$ in this context denotes the vector of predictor variables without the landmark variable $\varvec{V}$. The effect of $\varvec{V}$ is absorbed into $\lambda _0(u, \varvec{V})$. The development above leads to an analogue to the LA in terms of both model parameterization and estimation approach, with a change from landmark time to the more general concept of landmark variable. This change bypasses the use of the time since baseline when it does not have an explicit clinical interpretation. Both (1) and (3) are different parameterization of this more general bilateral relationship, and (3) includes (2) as a special case because the landmark time is part of $\varvec{Z}$. For this reason, we call (3) and its corresponding matching or weighting estimation the *generalized landmark analysis (GLA)*.

**Remark 1**. The matching idea is similar to the localized regression proposed by Kosel and Heagerty [13] (Note: not local polynomial regression), though that paper was written in the context of cross-sectional data but ours is for longitudinal data. The localized regression essentially matches training data with the subject in the validation data with respect to all predictors. However, such a matching approach is not feasible even with a small number of predictor variables in $\varvec{Z}$ unless the sample size is very large or a large caliper is allowed. To solve this problem, we have used a hybrid approach, which includes matching/weighting on the landmark variable(s) followed by a regression analysis using the other variables and the matched or weighted data.

**Remark 2**. In (3), the effect of $\varvec{V}$ is modeled nonparametrically, but the effects of other predictors in $\bar{\varvec{Z}}$ are modeled with linearity assumption. Therefore, there is less chance of model misspecification on $\varvec{V}$. This justifies selecting a strong prognostic variable in $\varvec{V}$, because presumably, misspecifying the effect of a stronger predictor has a larger effect on the prediction result. In the LA, the landmark time is usually a strong predictor when the baseline marks the start of a risk-changing period. In the GLA, there is often stronger predictors than the time since baseline because the latter does not have clinical meaning. For the CKD data application, the landmark variable can be the time-varying age or eGFR at clinical visits. The eGFR is the estimated glomerular filtration rate, an important biomarker for renal function. When matching on age at clinical visits, it is equivalent to the age-alignment method [19, 20], which was previously proposed for landmark analysis. However, it is widely known among nephrologists that eGFR is a much stronger predictor than age for terminal renal outcomes. In fact, the classification of CKD progression stages is solely based on eGFR values, not age. Broadly speaking, the GLA localizing on eGFR is equivalent to developing a prediction model tailored for the CKD stage of the new subject in validation dataset. This perhaps has better interpretation than GLA localizing on age (a tailored prediction model for the new subject’s age), given that eGFR is clinically more relevant.

**Remark 3**. Exact matching may not be feasible with continuous variables. Caliper matching is equivalent to kernel weighting with uniform kernel, unless it is restricted to the less efficient approach of selecting no more than one matched row per subject from the landmark training dataset. The implementation of GLA in this paper is based on kernel weighting. There could be more than one landmark variables in $\varvec{V}$, as illustrated by the WisARD data example below. From the kernel regression literature, it should be rare to use more than two landmark variables, unless the dataset is extremely large.

**Remark 4**. The GLA algorithm has a tuning parameter, i.e., the bandwidth (or caliper in the case of matching). When a very large bandwidth is used, it is equivalent to fitting the landmark dataset $\left\{ T_{ij}, \delta _{ij}, t_{ij}, \varvec{Z}_{ij} \right\}$ with a single survival model and assuming that the model parameters do not vary with the landmark variable. When a very small bandwidth is used, the model parameters are estimated with large sampling variation. Neither case is expected to produce optimal prediction. There is no evidence that the relationship between bandwidth and prediction accuracy takes a U-shape with a single optimal bandwidth in the middle. Therefore, we recommend calculating the cross-validated prediction accuracy with a range of reasonable bandwidth choices, and pick the one producing the best accuracy. This is illustrated in our data application below. If multiple bandwidths provide similar prediction accuracy in cross-validation, the smallest is preferred as long as the estimated parameters vary with the landmark variable. Loss of efficiency is another aspect of the bias-variance trade-off, but it is less of a concern when the prediction accuracy is similar. In practice, the distribution of the landmark variable may have some sparse regions. To deal with this issue, we can use an adaptive bandwidth choice, such as the span. We define the bandwidth as the $\gamma$th quantile of the distances between $\varvec{V}^*$ and all $\varvec{V}_{ij}$ in the training dataset. Here $\gamma \in (0, 1]$ is a pre-specified span parameter.

### Prediction accuracy assessment

Following the common practice in dynamic prediction literature [1, 2, 7, 15], we used two prediction accuracy measures in our data analysis, the area under the time-dependent ROC curve (AUC) [21] and time-dependent Brier score [22]. They quantify the discrimination and calibration of the predicted risk score, respectively. Let $\tilde{T}$ and $p^*$ denote the time-to-event outcome and the predicted probability of event occurring within the prediction horizon $\tau$. The time-dependent sensitivity (*Se*) and specificity (*Sp*) are defined as $Se(c) = P(p^* \geqslant c | \tilde{T} \leqslant \tau )$ and $Sp(c) = P(p^* < c | \tilde{T} > \tau )$, where *c* is the threshold for a positive or negative prediction. The time-dependent ROC curve is a plot of 1 - *Sp*(*c*) vs. *Se*(*c*) at all possible values of *c*. The AUC is the area under this curve. The time-dependent Brier score is defined as $E( 1\{ \tilde{T} \leqslant \tau \} - p^* )^2$. In the definitions above, the probability or expectation is defined on the distribution of $\{ \tilde{T}, p^* \}$ in the validation data. Various statistical methods have been proposed to estimate the AUC and BS as defined above, by accounting for censored $\tilde{T}$ in the validation dataset [21–23]. To guard against over-fitting, we used cross-validation in the numerical studies of this paper. We randomly split the original dataset into a training and validation dataset, each with half of the subjects. We fit the model using training data and calculated the prediction accuracy using validation data. We repeated the model-fitting and prediction accuracy assessment in 2,000 bootstrap samples extracted from the training data and validation data respectively, and then calculated the 95% bootstrap confidence interval. To further reduce sampling variability of this process, we repeated the split 5 times and the results were averaged.

## Statistical Analysis Results

### CRIC study

**Study description**. The Chronic Renal Insufficiency Cohort (CRIC) is a multi-center, observational longitudinal cohort study [24]. The CRIC dataset in this paper includes 3, 939 adult patients with chronic kidney disease (CKD), aged 21 to 74 years at enrollment. These CKD patients underwent annual in-person clinic visits to collect blood and urine specimens and assess mental and physical status. The most important biomarker for kidney function, the eGFR (estimated glomerular filteration rate, in mL/min/1.73$m^2$), was estimated at each clinical visit from serum creatinine, serum Cystatin C, age, gender, and race using the CKD-EPI equation [25]. At the time of study enrollment, the majority of the CRIC study participants had some renal dysfunction: 15.0% in CKD stage 1 and 2 (eGFR in 60-90), 66.5% in CKD stage 3 (eGFR in 30-60), and 18.5% in CKD stage 4 and 5 (eGFR $\le$ 30). Of note, the CRIC study is an observational study on a chronic disease, and the participants did not initiate any new intervention or have a risk changing event at the time of enrollment. In our dataset, most subjects had over 5 clinical visits, with a median of 7. About 9.4% subjects only had one visit.

For the purpose of illustrating the proposed statistical methodology, we define the outcome of interest as the composite clinical endpoint of ESRD and death, which is subject to right censoring. The predictors include eGFR, the urine protein to creatinine ratio (UPCR), age, and sex. With the exception of sex, the predictors are longitudinal and vary with the clinical visits. Table 1 presents the descriptive statistics of the cohort at baseline and by CKD stages. Since the clinical visits are the units of analysis in landmark modeling, and most predictors vary with clinical visits, the data are summarized in Table 1 by clinical visits instead of subjects. In the spirit of GLA, we did not present patient characteristics among the at-risk subjects defined by time since study enrollment.

**Table 1:** Characteristics of patients at study entry and stratified by CKD stages at the follow-up clinical visits. Chronic Renal Insufficiency Cohort Study, United States, 2003-2019

	All Follow-up	Baseline	CKD Stage 1-2	CKD Stage 3	CKD Stage 4-5
No. (%) Patients	3939 (100%)	3939 (100%)	1028 (15.0%)	3168 (66.5%)	1917 (18.5%)
No. Visits	28638	3939	4904	17388	6346
eGFR^a^	43.9 (17.0)	44.3 (15.0)	70.5 (9.36)	44.4 (8.33)	22.2 (5.57)
log UP/CR ^a^	-1.63 (1.48)	-1.49 (1.64)	-2.39 (1.05)	-1.81 (1.35)	-0.54 (1.50)
Age ^a^	62.7 (11.0)	57.7 (11.0)	57.4 (11.2)	63.9 (10.4)	63.4 (11.3)
Age ^b^	62.7 (11.0)	57.7 (11.0)	55.5 (11.6)	58.7 (10.5)	60.8 (11.4)
Sex ^b^					
Female	1778 (45.1%)	1778 (45.1%)	452 (44.0%)	1407 (44.4%)	861 (44.9%)
Male	2161 (54.9%)	2161 (54.9%)	576 (56.0%)	1761 (55.6%)	1056 (55.1%)
Age groups ^b^					
< 40	317 (8.05%)	317 (8.05%)	119 (11.6%)	203 (6.41%)	106 (5.53%)
[40,50)	493 (12.5%)	493 (12.5%)	159 (15.5%)	347 (11.0%)	206 (10.7%)
[50,60)	1169 (29.7%)	1169 (29.7%)	326 (31.7%)	900 (28.4%)	458 (23.9%)
[60,70)	1433 (36.4%)	1433 (36.4%)	326 (31.7%)	1257 (39.7%)	679 (35.4%)
$\geqslant 70$	527 (13.4%)	527 (13.4%)	98 (9.53%)	461 (14.6%)	468 (24.4%)
Time to event ^c^	11.3 (3.80)	8.84 (4.85)	12.9 (2.37)	11.7 (3.48)	8.61 (4.22)

^a^ Numbers are summarized at the clinical visit level.

^b^ Numbers are summarized at the patient level. For Age, the values are based on the first encounter in each cohort.

^c^ This is the mean (standard deviation) of residual follow-up in years since each clinic visit

At baseline, the majority of the patients were older adults, 1169 (29.7%) between 50-60 and 1433 (36.4%) between 60-70. The mean residual time-to-event is 8.84 years at baseline. During the follow-up, the patients were most often in CKD Stage 3 (17,388 visits from 3168 patients). Although the eGFR generally declines over time, nonlinear progression trajectories are also common and a patient may have both progression and regression among various CKD stages [26]. The logarithmic UPCR generally increases with the progression of CKD, from an average of -2.39 in CKD Stage 1-2 to -0.54 in stage 4-5. Older age is also correlated with CKD progression, from an average of 55.5 in CKD Stage 1-2 to 60.8 in CKD Stage 4-5. The gender proportions remain similar among different CKD stages.

**Fig. 1:**
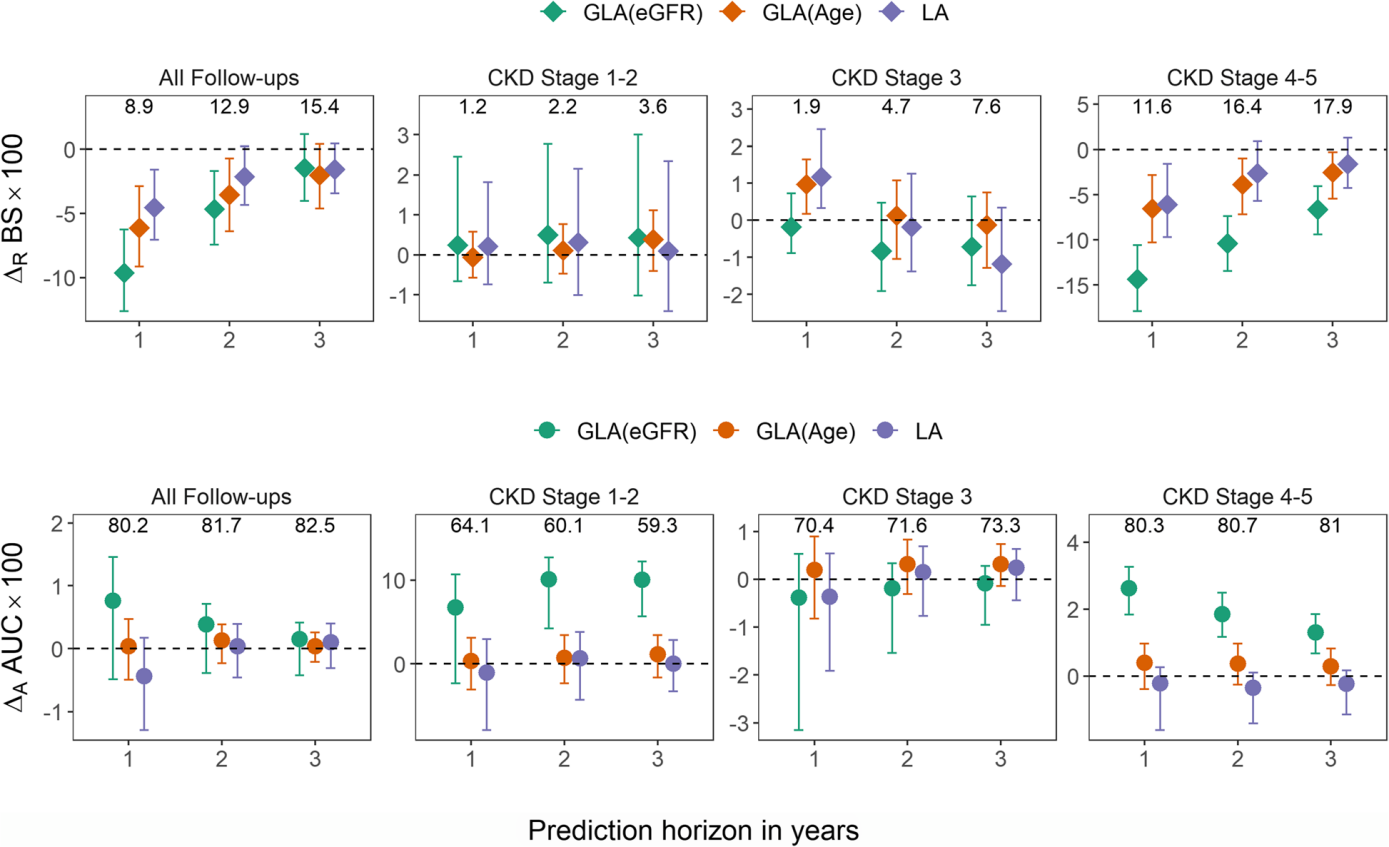
Relative differences in Brier score ($\Delta _R BS$; upper panel) and absolute difference in AUC ($\Delta _A BS$; lower panel) comparing GLA(eGFR), GLA(Age) and LA to SPM over (from left to right) a random sample of all follow-up observations, observations in CKD stages 1 and 2, observations in CKD stage 3, and observations in CKD stage 4 and 5 in CRIC study. The error bars represent 95% bootstrap percentile confidence intervals. The actual BS $\times 100$ and AUC $\times 100$ for the benchmark SPM are annotated on the top axis

**Fig. 2:**
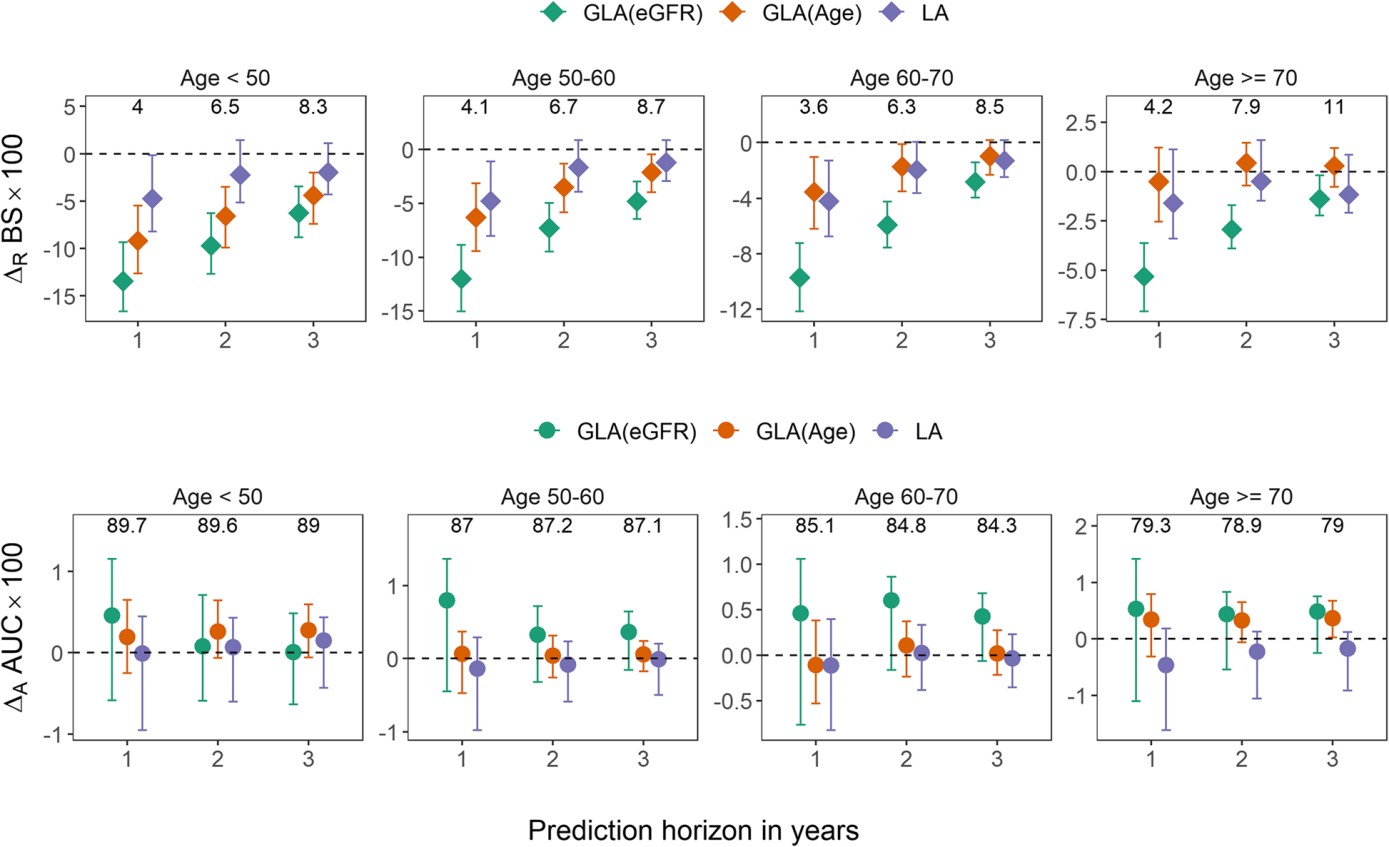
Relative differences in Brier score ($\Delta _R BS$; upper panel) and absolute difference in AUC ($\Delta _A BS$; lower panel) comparing GLA(eGFR), GLA(Age), and LA to SPM over (from left to right) observations that has age $< 50$, age in [50, 60), age in [60, 70), and age $\geqslant 70$ in CRIC study. The error bars represent 95% bootstrap percentile confidence intervals. The actual BS $\times 100$ and AUC $\times 100$ for the benchmark SPM are annotated on the top axis

**Fig. 3:**
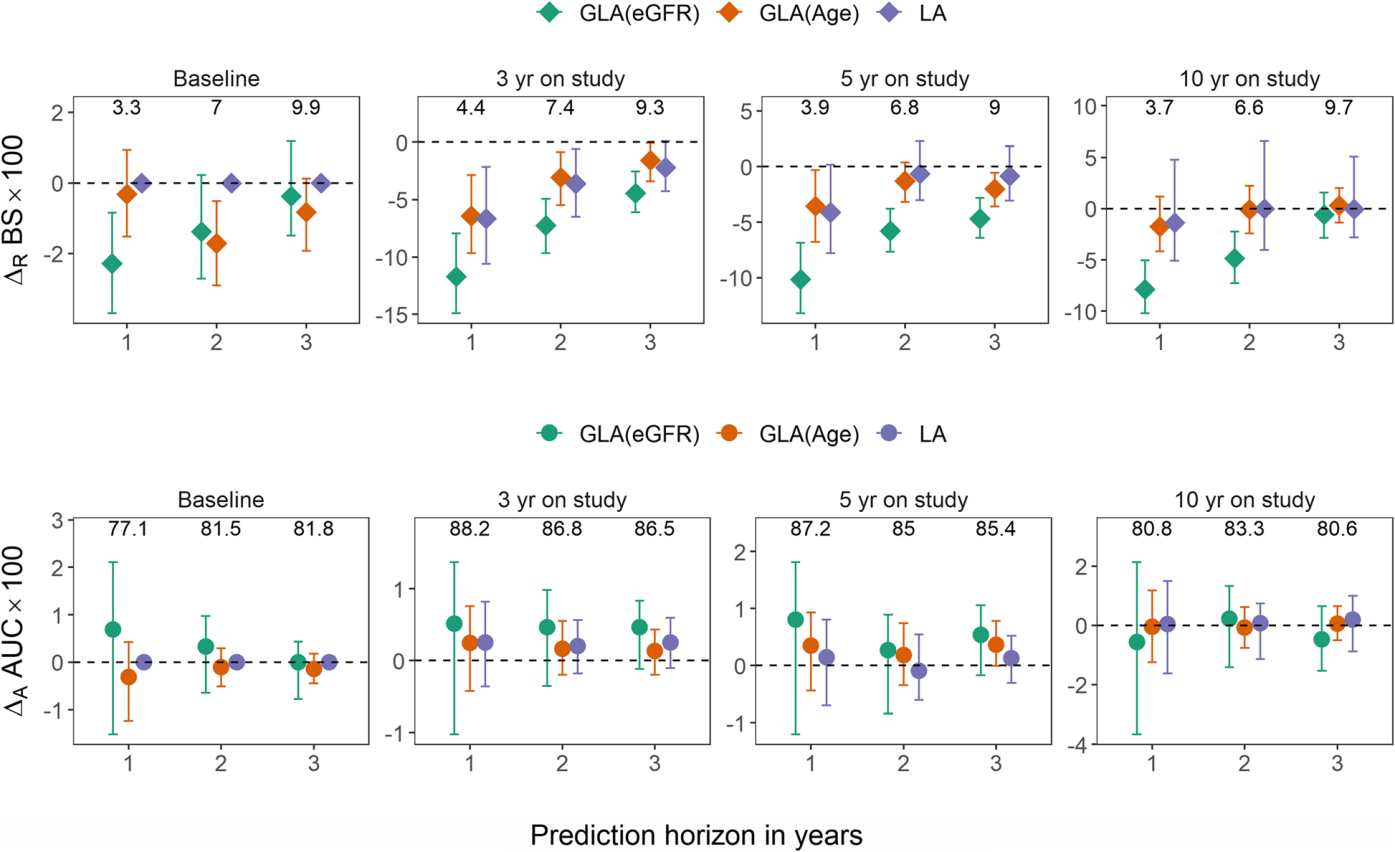
Relative differences in Brier score ($\Delta _R BS$; upper panel) and absolute difference in AUC ($\Delta _A BS$; lower panel) comparing GLA(eGFR), GLA(Age), and LA to SPM over (from left to right) observations at baseline (study entry time), 3-year on study, 5-year on study, and 10-year on study in CRIC study. The error bars represent 95% bootstrap percentile confidence intervals. The actual BS $\times 100$ and AUC $\times 100$ for the benchmark SPM are annotated on the top axis

**Statistical methods**. We implemented two GLA methods: a GLA localized on the landmark variable of eGFR, denoted by GLA(eGFR), and a GLA localized on the landmark variable of age, denoted by GLA(Age). Both eGFR and age are strong prognostic variables for the outcome, but eGFR is believed to have a more direct relationship from a clinical perspective. For comparison purposes, we also implemented the LA. It was equivalent to a GLA localized on the time since study enrollment and hence was denoted by GLA(time). We used the prediction accuracy of SPM as the benchmark, and compared the three landmark methods to it. For the time-dependent Brier score, we reported the relative difference from the benchmark, because the value of the Brier score is less informative than the difference in model comparison. For the time-dependent AUC, we reported the absolute difference from the benchmark, because the value of the AUC is informative. We considered a variety of validation datasets, defined either by all follow-up visits (Fig. 1), visits when the patients were in various CKD stages (Fig. 1), age groups (Fig. 2), or visits around various landmark times (Fig. 3). These validation datasets were created by using the aforementioned cross-validation procedure, eligibility criteria (e.g., CKD stages or age groups) applied as needed. These validation datasets were chosen because eGFR, age, and time since study entry were used as landmark variables in the GLA methods.

**Results**. Figures 1-3 show the prediction accuracy results of the three GLA and LA methods in comparison with the SPM. The relative performance among these three methods can also be seen on the plots. The plots are organized by various subpopulations of validation, and within each plot, the results are grouped according to the prediction horizon. Higher AUC and lower Brier score indicate better prediction accuracy. The Brier score is in general a more sensitive predictive performance measure than the AUC because its calculation uses the actual predicted probability values, while the AUC uses only their ranks.

First, GLA(eGFR) performed better than SPM and LA = GLA(time) in all scenarios in terms of Brier scores. Additionally, GLA(eGFR) performed better than GLA(age). GLA(eGFR), GLA(age) and GLA(time) all used longitudinal data to train their models, but they differed in model specification. Although the GLA model in (3), of which the LA model (2) was a special case, has a flexible formulation, some degree of misspecification is inevitable in real data such as CRIC. We argue in the Methods section that it is generally more advantageous to reduce misspecification of a strong predictor than a weak predictor, when it is not feasible to localize on both of them. The data analysis results supported that argument, because eGFR is widely known as the strongest predictor of CKD progression. Notably, the GLA(eGFR) has more improvement than other methods in CKD stages 1-2 and CKD stages 4-5. These are the two ends of the eGFR spectrum among the CRIC study subjects. Regardless of what the landmark variable is, Model (3) has a linear predictor structure in it, which is subject to model misspecification. A linear model usually has more extrapolation error near the boundaries of the covariate range. The extrapolation error of a strong predictor is more consequential than that of a weak predictor. The GLA(eGFR) nearly eliminates the extrapolation error of eGFR, due to the localization. This feature of GLA has important implications to its use in clinical practice. The prediction of ESRD is less useful for a CKD stage 3 patient, because that patient remains years away from reaching ESRD. But it is very important for patients with more advanced disease stages. Therefore, a risk prediction model that accurately predicts ESRD among the advanced CKD would be very helpful for the physician and patient to plan for dialysis or kidney replacement therapy.

Second, the SPM generally performed similarly or worse than the three landmark methods. The SPM was subject to model misspecification. It might also be applied out of its context in the sense that many validation datasets were extracted from the follow-up visits, which differed from the baseline data. GLA(age) and GLA(time) performed similarly. Age is a weak predictor and time since study entry is linearly related to age.

Third, the prediction horizon had little effect on the relative ordering of prediction accuracy results. These prediction horizons were chosen to avoid extending analysis beyond the range of follow-up. They are short compared to the natural history of CKD, but on the other hand, the advantage of dynamic prediction is to update the prediction adaptively as longitudinal data are collected. We speculate that there was perhaps little deviation from the proportional hazard assumption within these horizons, which contributed to the similarity of results. However, it is noteworthy that GLA(eGFR) appeared to have more improvement than SPM with shorter horizons. The ESRD to a great extent is triggered clinically by eGFR dropping below 10 to 15 mL/min/1.73m$^2$. Therefore, predicting ESRD is nearly equivalent to predicting future eGFR. With a shorter horizon, the association between eGFR and outcome was stronger, which helped GLA(eGFR), the method that modeled the effect of eGFR more appropriately.

**Fig. 4:**
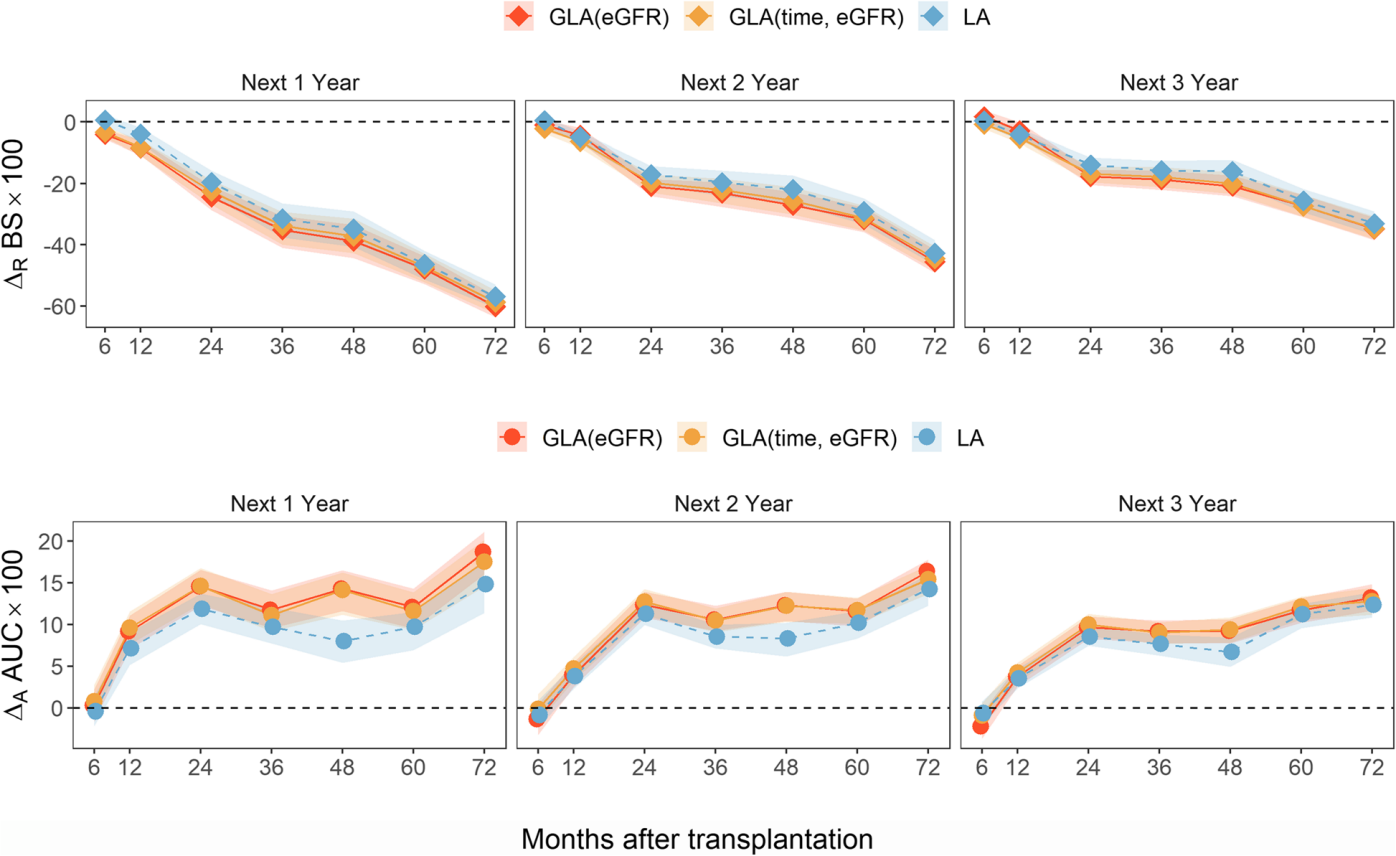
Relative differences in Brier score ($\Delta _R BS$; upper panel) and absolute difference in AUC ($\Delta _A BS$; lower panel) comparing GLA(eGFR), GLA(time, eGFR), and LA to SPM in predicting the composite outcome of graft failure and death in the next 1-, 2-, and 3-year at 6 to 72 months after transplantation in WisARD. The “time” denotes time-on-study. Baseline is the 6th month after kidney transplantation. Here, LA is equivalent to a GLA localizing on time-on-study only, i.e., GLA(time). The ribbons represent 95% bootstrap percentile confidence intervals

Fourth, if we ignore the conceptual issue with the baseline, and compare LA with the SPM, Fig. 3 shows that the former is always similar or better. This is not surprising. The validation datasets in that figure are defined from the at-risk population of selected landmark time, which always match the training datasets of the LA but do not in general match the training dataset of the SPM. The purple diamonds corresponding to the baseline validation dataset are at zero because in this scenario the LA is the same as SPM. A similar phenomenon has been noted by others [12]. However, it is worth noting that the improvement of LA over SPM was quite small over the landmark times of CRIC data. In fact, it is much smaller than the improvement in the landmark analysis of WisARD dataset described below (Fig. 4). As discussed in the Introduction, WisARD study has a clinically meaningful baseline, while CRIC does not. The dynamics of the at-risk population changed more prominently in WisARD. This supports the fundamental notion of this paper, that the concept of baseline is important in landmark modeling problems. When the baseline is not clinically meaningful, as in the CRIC, the LA not only encounters more difficulty with its interpretation, but also results in limited gain in predictive accuracy compared with SPM, despite more complicated modeling effort. The GLA(eGFR), in contrast, offers more improvement than LA, even when the validation datasets match the training datasets of LA (Fig. 3).

### WisARD study

**Study description**. The Wisconsin Allograft Recipient Database (WisARD) study is a registry of kidney transplant recipients at the University of Wisconsin Hospital and Clinics. Our data include patients who underwent transplantation between 1994 to 2013 and were followed to 2019. Prior to the renal transplantation, the information of the donor and recipients were gathered. After the transplantation, the clinical assessment, lab tests, various acute or chronic rejection events, and hospitalization episodes were recorded from various clinical visits. For our analysis, the outcome variable was a composite endpoint of death and graft failure. Graft failure was defined as re-transplantation or return to dialysis. The study population consisted of 3,784 patients who still had a functioning graft at 6 months after transplantation, which was treated as baseline in this analysis. The initial 6 months after kidney transplantation were excluded from analysis due to complicated post-surgical dynamics of the health conditions. Predictors include eGFR, the number of rejection in the past 12 months, age, and the donor status (living / deceased). With the exception of donor status, all other predictors are longitudinal and vary over time.

We summarized in Table 2 the characteristics of at-risk patients at 6 (baseline), 12, 24, and 36 months after transplantation. The time since baseline (landmark time) is a clinically meaningful quantity and prognostic factor for the patients, which justified the summary by this variable. Over time, the number of at-risk patients decreased from 3,784 to 2,707, as those who reached the composite endpoint or censoring were excluded from follow-up. Therefore, the relatively stable mean eGFR and mean age reflected both within-individual change in these variables as well as a change in the population. Among the remaining at-risk patients, the frequency of acute and chronic rejection appeared to increase over time, and the residual follow-up time was reduced as expected.

**Table 2:** Characteristics of patients at baseline (6 month post-transplantation), and 1-, 2-, 3-, and 4-year after renal transplantation. Wisconsin Allograft Recipient Database

	Baseline	1 Year	2 Year	3 Year	4 Year
No. Patients	3893	3154	2525	2012	1668
eGFR	55.6 (18.6)	54.9 (18.6)	56.9 (19.8)	56.6 (20.2)	56.1 (19.8)
Age	51.6 (12.9)	52.4 (12.8)	53.5 (12.6)	54.6 (12.6)	55.1 (12.6)
Number of acute rejections in the past (at most) 12 months:					
0	3237 (83.1%)	2542 (80.6%)	2404 (95.2%)	1958 (97.3%)	1632 (97.8%)
$\ge$1	656 (16.9%)	612 (19.4%)	121 (4.79%)	54 (2.68%)	36 (2.16%)
Number of chronic rejections in the past (at most) 12 months:					
0	3 (0.08%)	12 (0.38%)	9 (0.36%)	11 (0.55%)	6 (0.36%)
$\ge$1	3237 (83.1%)	2542 (80.6%)	2404 (95.2%)	1958 (97.3%)	1632 (97.8%)
Number of hospitalization in the past (at most) 12 months:					
0	2372 (60.9%)	1765 (56.0%)	1972 (78.1%)	1664 (82.7%)	1402 (84.1%)
$\ge$1	1521 (39.1%)	1389 (44.0%)	553 (21.9%)	348 (17.3%)	266 (15.9%)
Status of Donor:					
Deceased	2365 (60.8%)	1885 (59.8%)	1480 (58.6%)	1170 (58.2%)	941 (56.4%)
Living	1528 (39.2%)	1269 (40.2%)	1045 (41.4%)	842 (41.8%)	727 (43.6%)
Time to event ^a^	69.7 (50.2)	69.8 (48.8)	66.8 (46.1)	62.9 (43.4)	58.7 (40.6)

^a^This is the mean (standard deviation) of residual follow-up time in months, averaged over clinic visits

**Statistical method**. The statistical analysis was similar to the CRIC data. However, since there is a clear baseline, the validation datasets were defined by landmark time, as in typical landmark modeling. We studied and compared four methods: SPM, LA = GLA(time), GLA(eGFR), and GLA(time, eGFR). The fourth method was Model (3) with a vector of two landmark variables in $\varvec{V}$, the landmark time and eGFR. They are both important prognostic variables. The estimation was done using bivariate kernel weights. GLA(age) and GLA(eGFR) were not considered here because the landmark time is equivalent to graft survival at the time of prediction, one of the most clinically relevant prognostic factors among graft recipients. The validation set is selected as the population still at risk of graft failure or death at 6-month (baseline), and at 1- to 6-year after transplantation (landmark times, or simply “time”). For each patient in the validation set, GLA identifies a neighborhood of data around the landmark time and the current measured value of eGFR of this specific patient from the training set, on which the predicted risk is calculated. Simultaneously localizing on landmark time and eGFR makes it necessary to use a larger neighborhood due to the “curse of dimensionality”. Thus, both time and eGFR were further linearly adjusted in the GLA model as covariates. The cross-validation procedure and comparison of prediction accuracy between SPM and GLA were carried out in the same way as in the CRIC data analysis.

**Results**. The result is presented in Fig. 4. First, the LA performed substantially better than SPM, as expected from typical landmark modeling. Second, both GLA(eGFR) and the GLA(time, eGFR) resulted in further improvement over LA, possibly because GLA had less chance of model misspecification. GLA(eGFR) performed similarly to GLA(time, eGFR). The eGFR, an important biomarker of renal function, is also a good prognostic biomarker of future graft failure. When eGFR is known, the time on graft provides little additional prognostic information. In this paper, we focused our discussion on situations without an explicit baseline, and demonstrated the interpretation and predictive advantages of GLA in that situation. Figure 4 shows that in situations with an explicit baseline, the concept of GLA can be used to further improve the performance of LA.

## Discussion

GLA can be viewed simultaneously as a matching algorithm, a kernel weighting procedure, and a model specification that allows the parameters to vary with one or more landmark variables. It is analogous to, and generalizes the conventional LA. The GLA has better interpretation than the LA when there is no explicit baseline in the data. Our data analysis show that the GLA may produce better prediction accuracy than the LA in such situation (e.g., CRIC) and also in situations with explicit baseline (e.g., WisARD). We attribute this phenomenon to the model flexibility and specification. Compared with the LA, GLA offers a different, and more general, model specification for the bilateral relationship between predictors and residual survival at each clinical visit. This model specification allows all the model parameters to be functions of landmark variables, which is equivalent to a matching or weighting algorithm that localizes on the landmark variables. Consequently, the misspecification of the effect of landmark variables is nearly eliminated. On the one hand, this feature helps the GLA to outperform LA in the data without an explicit baseline, because the landmark variable in GLA is a strong predictor, while the landmark variable in LA is a weak or null predictor in this situation. Misspecifying a strong predictor is expected to have a larger effect on prediction accuracy. On the other hand, this feature also helps the GLA to further improve upon the LA by localizing on other strong predictors in addition to the landmark time, in situations where the data have an explicit baseline. Both GLA and LA performed similarly or better than SPM in almost all situations considered in our two data applications. This result highlights the importance of considering dynamic prediction when the risk prediction model is developed from longitudinal data.

As GLA does not require that the landmark variable be tied to the time since baseline, it allows more readily interpretable results in other clinical contexts. Our results suggest that the flexibility to choose localization based on a stronger predictor, such as eGFR rather than age in our example, may result in better prediction. This may be especially advantageous in kidney transplantation, in which frailty or other markers of health status may provide more relevant predictive information than does biological age [27]. The proportion of new kidney transplant recipients > 65 years old in the US more than doubled from 2000 to 2018, highlighting the diminishing reliance on definitive age limits and the need for alternative predictors of outcomes [28]. As markers of health status also are potentially modifiable, improved prediction may help guide clinical decision-making both before and after transplantation.

The main conclusions were drawn from the analysis of two real data examples. While this may limit the generalizability of the conclusions, we have presented methodological arguments to explain the findings. We chose not to use simulated experiments because it is a widely recognized difficulty to simulate data while ensuring that the landmark model is correctly specified at all landmark times [1, 29]. This is because the landmark model, regardless (1) or (3), is not a single model with varying coefficients but a collection of working models indexed by the landmark variable. If we simulated longitudinal and survival data from a joint distribution without guaranteeing that GLA, LA or SPM work under correction specification, the results would be difficult to interpret and may heavily depend on the simulation setting. Nonetheless, GLA needs to be studied further in other datasets and patient populations to establish the generalizability of the conclusions in this paper.

In the data analysis, we used the basic model form for all of SPM, LA and GLA in the sense that the covariates other than the landmark variables are additive in the model’s linear predictor. This is a conventional approach in practice. Since the better performance of the GLA is caused by reduced chance of model misspecification, it is possible to improve LA or SPM by using a more flexible model form such as adding nonlinear terms. However, a few issues are worth noting. First, GLA itself is a simple solution to make LA more flexible. This is shown in the WisARD data example. Second, all three models (GLA, LA, SPM) can be made more flexible. But the alternative model formulations for each are extensive, which makes comparison difficult. We have hence resorted to the simplification of using the basic formulation, which is linear and additive in the covariates. Third, while the SPM can be made more flexible, it still has the critical drawback of using only the baseline data. In typical longitudinal cohort studies, the inclusion and exclusion criteria often limit the baseline population to be younger with earlier disease stages. The population tends to become older with more advanced diseases during the follow-up. Since the intended application of the risk prediction model is usually during the follow-up, this mismatch between training and validation data could affect the performance of SPM. In situations where the baseline visits can be viewed as a “snapshot” of all the longitudinal clinical visits (e.g., the electronic health records), the SPM would produce valid prediction. However, the SPM may not be the optimal solution even with flexible model formulation, because the majority of the longitudinal clinical visits are not used. The GLA and LA methods do not have such a problem and they come with automatic protection of misspecifying the effect of landmark variables. They can also incorporate predictive features of longitudinal history by design, which SPM cannot.

Left truncation may be an intrinsic issue in observational cohort studies of chronic disease, and it causes bias to estimating the population distribution [30]. Those who had the outcome event early in life were less likely to be included in the data. In prediction problems, however, this does not affect the predictive accuracy as long as the training and validation data are both subject to left truncation. It is worth further research when the left truncation distributions differ in training and validation datasets.

Our work in this paper highlights the importance of reducing model misspecification in landmark modeling. Future work should be geared toward developing model checking procedures and more flexible modeling approaches. GLA and LA are not a single model with varying coefficients, but a collection of many working models. The model checking and fitting procedures developed for a single statistical model should be adapted to properly address this challenge.

## Conclusion

GLA generalizes the LA by broadening the concept of the landmark variable so that it does not have to be the time since baseline. This change leads to better interpretation when the baseline is not a risk-changing clinical milestone. GLA can be viewed as a matching algorithm, weighted estimation, or a varying coefficient model for residual survival that has a more flexible formulation than the LA. The flexibility and adaptivity to the validation population may help produce more accurate prediction.

## Data Availability

The CRIC study data are publicly available through a request to the Central Repository of National Institute of Diabetes and Digestive and Kidney Diseases. Requests for de-identified data from WisARD should be directed to Dr. Astor at bcastor@medicine.wisc.edu.
